# Antifungal, Antioxidant and Antibiofilm Activities of Essential Oils of *Cymbopogon* spp.

**DOI:** 10.3390/antibiotics11060829

**Published:** 2022-06-20

**Authors:** Wafa Rhimi, Mona A. Mohammed, Aya Attia Koraney Zarea, Grazia Greco, Maria Tempesta, Domenico Otranto, Claudia Cafarchia

**Affiliations:** 1 Dipartimento di Medicina Veterinaria, Università degli Studi di Bari, 70010 Valenzano, Italy; wafa.rhimi@uniba.it (W.R.); aya.zarea@uniba.it (A.A.K.Z.); grazia.greco@uniba.it (G.G.); maria.tempesta@uniba.it (M.T.); domenico.otranto@uniba.it (D.O.); 2 Medicinal and Aromatic Plants Research Department, Pharmaceutical and Drug Industries Institute, National Research Centre (NRC), Dokki, Giza 12622, Egypt; monaarafamohammed@yahoo.com; 3 Department of Microbiology and Immunology, Veterinary Research Institute, National Research Centre (NRC), Dokki, Giza 12622, Egypt; 4 Faculty of Veterinary Sciences, Bu-Ali Sina University, Hamedan 65175, Iran

**Keywords:** antifungal, antioxidant, antibiofilm, *Cymbopogon citratus*, *Cymbopogon proximus*, essential oils

## Abstract

Essential oils (EOs) of *Cymbopogon citratus* and *Cymbopogon proximus* are known as sources of monoterpenes and sesquiterpenoids, although their biological activities have not been well investigated. In this study, the compositions of *C. citratus* and *C. proximus* EOs of Egyptian origin and their antifungal and antibiofilm properties against *Candida* spp. and *Malassezia furfur* were investigated. Antioxidant activities were also evaluated. GC-MS showed the presence of nine and eight constituents in *C. citratus* and *C. proximus* EOs, respectively, with geranial and neral as the major compounds of *C. citratus* EO and piperitone and α-terpinolene as the major compounds of *C. proximus* EO. Both EOs showed antifungal (MIC values ranging from 1.25 to 20 µL/ mL) and antibiofilm activities (% of reduction ranging from 27.65 ± 11.7 to 96.39 ± 2.8) against all yeast species. The antifungal and antibiofilm activities of *C. citratus* EO were significantly higher than those observed for *C. proximus* EO. *M. furfur* was more susceptible to both EOs than *Candida* spp. Both EOs exhibited the highest antioxidant activity. This study suggests that *C. citratus* and *C. proximus* EOs might be an excellent source of antifungal, antibiofilm and antioxidant drugs and might be useful for preventing *Malassezia* infections in both medical and veterinary medicine.

## 1. Introduction

Essential oils (EOs) are secondary metabolites and organic compounds with a low molecular weight produced by plants [1]. They play a role as regulators of cell metabolism under environmental stress and pathogenic attacks and are considered to be relevant therapeutic drugs for the treatment of animals and human infectious diseases [2,3]. In particular, EOs may represent sources of bioactive agents with a large spectrum of pharmacologic applications (i.e., antiphlogistic, spasmolytic, antinociceptive and antioxidant activities) [4]. In addition, their antimicrobial and antibiofilm activities stimulated the interest of the scientific community in proposing a solution for alarming multidrug resistance phenomena [5]. 

Several EOs displayed fungicidal and antibiofilm effects against different fungal pathogens, namely *Aspergillus*, *Candida*, *Cryptococcus* and *Fusarium*, which represent the major causes for human and animal diseases with high mortality, mainly in immunocompromised patients [6,7,8]. Recently, *Malassezia* yeasts have emerged as a threat to both human and veterinary medicine. These yeast species are known to cause skin disorders and fungemia in immunocompromised patients [9]. Despite attempts to control such yeast infections with topical and systemic antifungals, recurrence of clinical signs of skin infections, as well as treatment failure in preventing or treating *Malassezia furfur* fungemia, have been reported, most likely due to the occurrence of resistant phenomena. Interestingly, essential oils are proposed as promising candidates to control or to prevent *Malassezia*-associated skin diseases both in humans (i.e., atopic dermatitis, dermatitis, pityriasis versicolor and *Malassezia folliculitis*) and in animals [9,10,11,12].

In particular, biofilm formation is one of the mechanisms related to multidrug resistance phenomena associated with the highest lethality of infected patients [13]. Several antifungal agents (e.g., amphotericin B, fluconazole, flucytosine, itraconazole and ketoconazole) fail to treat infections caused by yeasts forming biofilm [13], thus raising scientific efforts for the selection of new molecules. EOs have proven to be more effective against fungal biofilm than conventional drugs due to their high content of monoterpenes and sesquiterpenes [14]. Monoterpenes and sesquiterpenes have the potential to affect membrane integrity and suppress genes related to biofilm formation [15]. Earlier studies have shown a large variability in the monoterpenes and sesquiterpenoids composition of Mediterranean medicinal plants EOs (e.g., *Origanum vulgare* L. (oregano), *Salvia officinalis* L. (sage) and *Thymus vulgaris* L. (thyme) (*Lamiaceae*)). Among Mediterranean plants, the genus *Cymbopogon* has been largely recommended for its high monoterpene and sesquiterpenoid content [16,17]. In particular, EOs of *Cymbopogon citratus* and *Cymbopogon proximus*, commonly named lemongrass and halfabar and largely diffused in Egypt, are used in traditional medicines as anti-diabetic, antihypertensive, antioxidant and anti-inflammatory drugs [18]. Although some investigations on the chemical and biological profiles of these species proved the presence of monoterpenes and sesquiterpenoids [19,20,21], there is a lack of scientific evidence regarding the chemical composition of Egyptian *C. citratus* and *C. proximus* EOs and their usefulness as antifungal, antibiofilm and antioxidant drugs. Thus, the present study was designed to characterize the composition of the EOs from *C. citratus* and *C. proximux* and to evaluate their antifungal, antibiofilm and antiradical properties. 

## 2. Results

### 2.1. Chemical Composition of C. citratus and C. proximus Essential Oils

**Figure 1:**
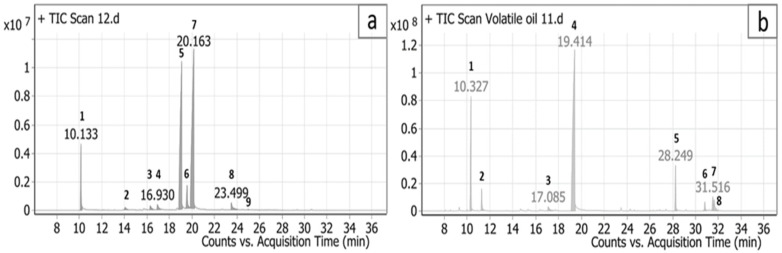
Total ion current (TIC) chromatogram of the volatile oil of *Cymbopogon citratus* (**a**) and *Cymbopogon proximus* (**b**) (The peak numbers are described in Table 1 and Table 2).

Extraction of *C. citratus* and *C. proximus* leaves by hydro-distillation produced EOs with yields of 3 and 3.75% (*v*/*w*), respectively. Their GC/MS profiles and chemical compositions (i.e., mass fragmentation and retention indexes) are presented in Figure 1 and Table 1 and Table 2.

**Figure 2:**
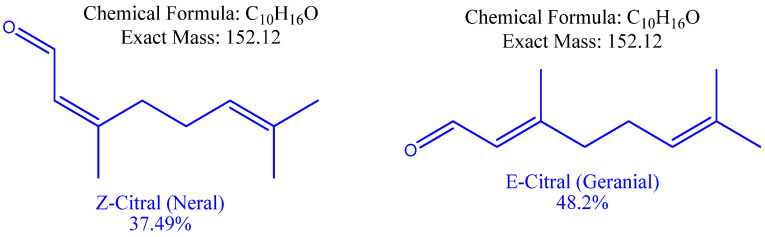
Chemical structure of neral and geranial as the major constituents of Egyptian *C. citratus* EO.

Monoterpenes were the most abundant compounds of both EOs representing 87.0 and 99.9% of the total oil composition. A total of nine compounds representing the whole bulk of *C. citratus* EO were identified, with geranial (48.2%) and neral (37.49%) being the major compounds (Figure 2). Eight compounds, representing 100% of *C. proximus* EO, were identified, with piperitone (66.99%) and α-terpinolene (15.7%) being the major compounds.

### 2.2. Antifungal Activity

The antifungal activities of *C. citratus* and *C. proximus* EOs obtained by the broth microdilution method are reported in Table 3. The minimal inhibitory concentration (MIC) and the minimal fungicidal concentration (MFC) values vary according to the EO’s origin. The MIC and MFC values of *C. citratus* EO were lower (MIC values from 1.25 to 5 µL/mL) than those registered for *C. proximus* EO (MIC values from 2.5 to 20 µL/mL). The *M. furfur* strains were the most sensitive species to both EOs. Among *Candida* spp., *C. catenulata* and *C. guilliermondii* were less sensitive to *C. citratus* EO.

### 2.3. Inhibitory Effects of Cymbopogon citratus and Cymbopogon proximus EOs on Candida *spp.* and Malassezia furfur Biofilms

**Figure 3:**
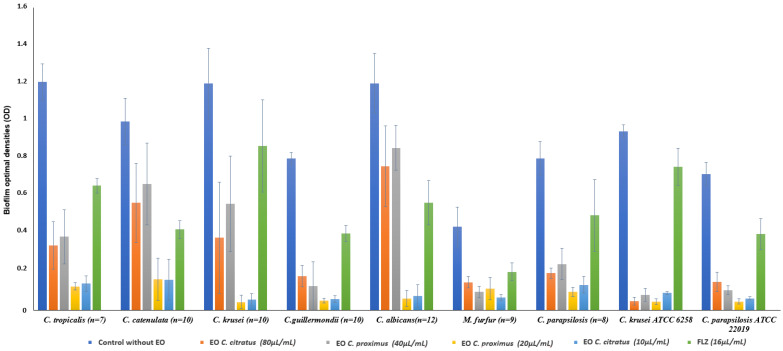
Absorbance of *Candida* spp. and *M. furfur* biofilm with and without *C. citratus*, *C. proximus* EOs and FLZ. *C. citratus* and *C. proximus* EOs were tested at different concentrations.

The XTT [2,3-bis(2-methoxy-4-nitro-5-sulfo-phenyl)-2H-tetra-zolium-5-carboxanilide] reduction assay showed that all tested yeasts were able to form biofilm within 24 to 48 h. Among the tested yeast species, *C. tropicalis* strains were the highest biofilm producers, whereas *M. furfur* strains were the lowest. All *Candida* spp. strains were higher biofilm producers (*p* < 0.05) than *M. furfur* strains. A significant decrease in biofilm formation compared to the control was observed in the tested yeast strains when grown in the presence of *C. citratus* or *C. proximus* EOs (Figure 3), showing an inhibition percentage ranging from 27.65 ± 11.7 to 96.39 ± 2.8%. The antibiofilm properties of both *C. citratus* and *C. proximus* EOs were significantly higher than those registered with FLZ (percentage of biofilm inhibition ranging from 19.68 ± 13.1% to 57.22 ± 5.3; Table 4). 

The antibiofilm effects of both EOs were not related to their concentrations. The *C. citratus* EO exhibited significantly higher anti-biofilm activity than *C. proximus* EO (74.01 ± 11.5 to 96.39 ± 2.8% vs. 27.65 ± 11.7 to 96.39 ± 2%) against all tested yeast species except *C. parapsilosis*, *M. furfur* and *C. krusei* ATCC 6258 strains. 

### 2.4. In Vitro Antioxidant Activity of C. citratus and C. proximus EOs

**Figure 4:**
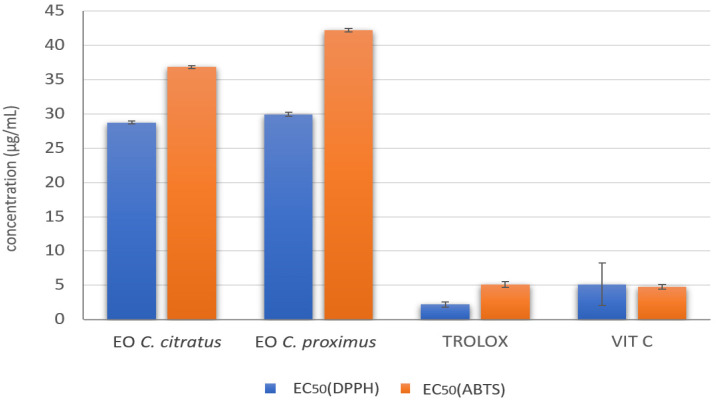
Radical scavenging activity against 2,2-Diphenyl-1-(2,4,6-trinitrophenyl) hydrazy (DPPH) and 2, 2′-Azino-Bis-3-Ethylbenzothiazoline-6-Sulfonic Acid (ABTS) of the *C. citratus* and *C. proximus* EOs.

DPPH and ABTS radicals can accept an electron or hydrogen radical to become stable radicals. They lose absorption when accepting an electron or hydrogen radical [22], which results in a visually noticeable discoloration and indicates the ability of the EOs to act as free radical scavengers or hydrogen donors [22]. *C. citratus* and *C. proximus* EOs showed high radical scavenging abilities for DPPH and ABTS. The effective concentration at which 50% of the DPPH or ABTS radicals were scavenged (EC_50_) ranged from 28.73 to 42.18 µg/mL (Figure 4). No statistically significant differences were registered between *C. citratus* and *C. proximus* EOs in scavenging DPPH and ABTS. Trolox and vitamin C demonstrated higher scavenging activity for DPPH and ABTS than those registered for EOs.

## 3. Discussion

The results of this study confirm that *C. citratus* and *C. proximus* are sources of terpenes and demonstrate that these EOs may represent excellent sources of antifungal, antioxidant and antibiofilm drugs. Interestingly, this study revealed for the first time the antifungal and antibiofilm activities against new and emerging yeast pathogens such as *C. catenulata, C. guilliermondii* and *M. furfur*. In particular, the chemical profiles of the EOs reveal the usefulness of both plants as sources of terpenes, as previously suggested [14]. In addition, since the yield of the EOs varies according to the plants, the results of this study suggest that *C. citratus* represents the better source for these compounds, thus confirming previous studies in which the yield of EOs of *C. citratus* and *C. proximus* of different origins (Burkina Faso, México, Algeria, and Egypt) were evaluated [23,24,25,26].

Since the EO content and composition can be considerably affected by the geographical origin, in this study, the yield of EO of *C. citratus* was higher than those previously retrieved in the same plants of different origins with the geranial and neral as major compounds [19,23,24,26].

Both *C. citratus* and *C. proximus* EOs displayed growth inhibition activity against yeasts. This finding is in line with previous studies investigating different medicinal plants presenting a richness of terpenes (i.e., *Origanum vulgare*, *Coriandrum sativum* L., *Juniperus communis* L., *Lavandula angustifolia* Mill, *Mentha arvensis* L., *Mentha pulegium* L., *Ocimum basilicum* L.) [27].

Compared to *C. proximus* EO, the highest antifungal activity displayed by *C. citratus* EO could be related to its higher monoterpene content, including geraniol. Furthermore, the richness of geraniol in *C. citratus* EO might also cause destabilization of fungal cell membranes. In this sense, earlier studies have revealed the potent antifungal activity of geraniol at concentrations ranging from 30 to 130 µg/mL against *Candida* spp. due to its ability to disrupt cell membrane integrity by interfering with ergosterol biosynthesis and inhibiting the very crucial PM-ATPase [28,29]. Moreover, the high MIC of *C. proximus* EO herein observed is in accordance with the moderate activity of piperitone against *Candida* spp. [30]. These results confirm the studies previously performed on some yeast species using the same plants of different origins. Particularly, the results of this study confirm previous findings about the inhibitory effect of *C. citratus* EOs from France and Brazil against some clinical *Candida* spp. (i.e., *C. albicans*, *C. krusei*, *C. tropicalis*, *C. glabrata*, *C. parapsilosis* and *C. tropicalis*) [31] and extend the broad spectrum of antifungal activities to other rare opportunistic fungal pathogens, such as *C. catenulata*, *C. guilliermondii* and *M. furfur*. However, the MIC values herein registered for *C. citratus* EO against *Candida* spp. were slightly higher than those registered for *C. citratus* EO from Asia and lower than those for *C. citratus* EO from Brazil [32,33], suggesting that the chemo-geographical variation in *C. citratus* EO might also affect its antifungal activities [34,35]. On the contrary, the antifungal activity of *C. proximus* EO in this study is in contrast with previous studies in which only antibacterial activities were detected [19] and might be due to the low dose of EOs previously employed (i.e., 0.25 to 1 μL/mL vs. 2.5 to 20 μL/mL in our study). 

Interestingly, *M. furfur* strains seem more susceptible than *Candida* spp. to both EOs and this might be due to the lipid capsule composition of *Malassezia* spp. that might favor EO solubilization, thus affecting their efficacy [36]. These findings propose that these EOs could be considered an effective alternative approach for the treatment of *M. furfur* skin infections, which are usually characterized by recurrences.

In particular, new guidelines for the treatment of these infections in animals propose the use of EOs as prophylactic procedures to decrease the risk of recalcitrant *Malassezia* spp. infection [37,38]. In addition, since these yeast species are considered emerging threats for immunocompromised patients (i.e., preterm infants), accurate hygiene of medical operators’ hands and incubators was usually required to prevent fungemia [39]. However, the chemical substances used for hygiene have very low efficacy against these yeast species; thus, EOs might be considered sources of active drugs for preventing strategies of *Malassezia* spp. systemic infections [40].

At present, this study demonstrated for the first time that *C. citratus* and *C. proximus* EOs are effective agents against biofilm formation. Anti-biofilm activities were also previously demonstrated for other EOs, including citronella, cinnamon, cascarilla bark and helichrysum [41], but the number of compounds with anti-biofilm effects are still scant and new molecules are requested. The excellent ability of *C. citratus* and *C. proximus* EOs to interfere with the mature biofilm of yeasts might be due to the hydrophobic interactions of monoterpenes with attachment forces such as lifshitz-Van der Waals, Brownian, sedimentation and electrostatic interaction forces, which are useful for yeast attachment to different surface types [42].

Interestingly, in this study, the antibiofilm activity should also be related to the antioxidant activities of *C. citratus* and *C. proximus* EOs. Indeed, both EOs, at very low concentrations, showed radical activities scavenging DPPH and ABTS in vitro (50%) comparable to those of synthetic antioxidants (i.e., butylated hydroxytoluene -BHT), possibly due to the high content of monoterpenes activities [43]. In particular, monoterpenes are able to absorb or neutralize free radicals due to their phenolic structure and redox properties [44,45]. In fungal cells, monoterpenes might act as pro-oxidants by disturbing the healthy redox cycle that might lead to an accumulation of reactive oxygen species (ROS) (i.e., hydrogen peroxide, superoxide and hydroxyl radicals) [46]. Usually, a healthy redox cycle promotes microbial attachment, thus favoring biofilm formation [39]. Inversely, in the presence of pro-oxidant compounds, a high level of ROS might favor a reduction of the extracellular polymeric substance (EPS) production, thus affecting the homogeneous structure, yeast numbers, and community composition of biofilm [46,47,48]. Recently, a strong association between oxidative stress and biofilm formation of bacteria and some yeast species has been demonstrated (*C. albicans*, *C. glabrata*, *C. krusei,* and *C. parapsilosis*) [47,49]. In detail, in *C. albicans* cells, the polyphenols from plants (i.e., magnolol and honokiol) induce ROS accumulation, causing decreased expression levels of specific genes (i.e., Ras-like protein 1-RAS1, enhanced filamentous growth protein -EFG1, Ty-transcription activator-TEC1, and ATP pyrophosphate-lyase-CDC35) involved in adhesion, yeast hyphal transition and biofilm formation [50]. Similarly, compounds that could target oxidative stress regulators, including antioxidants, could potentially be exploited as novel strategies for biofilm control [46]. However, the significantly higher antibiofilm activity of *C. citratus* EO compared to *C. proximus* EO might be attributable to the occurrence of specific components, mainly geranial and neral or to their synergistic activity, thus suggesting that the antibiofilm activities of EO might be due to different factors acting synergistically and/or additionally. In particular, it has been shown that geraniol is involved in the deterioration of the mature biofilm by affecting chitin and β-glucan synthesis, which are the major fungal cell wall components [51]. In addition, geranial and neral might act in synergy by decreasing intracellular adenosine triphosphate (ATP), pH and cell membrane integrity [52]. 

## 4. Materials and Methods

### 4.1. Plant Material and Essential Oil Isolation

*C. citratus* and *C. proximus* were collected from Siwa Oasis, governorate Nubian and Aswan governorate, Egypt, respectively, during September 2020. The plant species were identified by Dr. Monier Abd El-Ghani, Department of Taxonomy, Faculty of Science, Cairo University. Leaves of *C. citratus* and *C. proximus* were washed, dried in the shade, crushed into small pieces and 100 g were subjected to hydro distillation for 4 h. EO extraction was repeated 4 times. *C. citratus* and *C. proximus* EOs were extracted by steam distillation using a Karlsruhe apparatus. The resulting EOs were dried over anhydrous sodium sulfate and stored at −20 °C until their use. The EO concentrations tested for antifungal and antibiofilm activities ranged from 0.015 to 80 µL/mL being lower than those causing acute toxicity phenomena causing acute toxicity phenomena [24,53,54]. 

### 4.2. Identification of the Chemical Composition of EOs by Gas Chromatography–Mass Spectrometry Analysis (GC-MS)

The GC-MS system (Agilent Technologies) was equipped with a gas chromatograph (7890B) and mass spectrometer detector (5977A) at the Central Laboratories Network, National Research Centre (NRC), Cairo, Egypt. EOs were diluted with hexane (1:19, *v*/*v*). The GC-MS was equipped with an HP-5MS column (30 m × 0.25 mm internal diameter and 0.25 μm film thickness). Analyses were carried out using helium as the carrier gas at a flow rate of 1.0 mL/min at a split 1:30, injection volume of 1 µL at the following temperature program: 40 °C for 1 min; rising at 4 °C /min to 150 °C and held for 6 min; rising at 4 °C/min to 210 °C and held for 1 min. The injector and detector were held at 280 °C and 220 °C, respectively. Mass spectra were obtained by electron ionization (EI) at 70 eV, using a spectral range of m/z 50–900 and a solvent delay of 5 min. The identification of different constituents was determined by comparing the spectrum fragmentation pattern with those stored in the Wiley and NIST Mass Spectral Library. 

### 4.3. Antifungal Activities

#### 4.3.1. Yeast Strains

A total of 68 strains isolated from the cloaca of domestic and wild animals or from the skin of hospitalized human patients with *M. furfur* fungemia were employed for antifungal testing (Table 5). The strains were identified biochemically and molecularly, as previously reported [55]. All strains were obtained from the fungal collection of the Department of Veterinary Medicine at the University of Bari Aldo Moro, Italy.

#### 4.3.2. Antifungal Activity

The minimal inhibitory concentration (MIC) and the minimal fungicidal concentration (MFC) of EOs were determined by broth microdilution methods according to the CLSI protocol for *Candida* and the CLSI modified protocol for *Malassezia,* as previously reported [56,57]. Stock inoculum suspensions of *Candida* spp. and *M. furfur* were adjusted to an optical density of 0.5–2.4 McFarland, respectively, equivalent to 5 × 10^6^ colony forming units (CFU)/mL. Two serial dilutions of *Candida* spp. (1:10 *v*/*v*) and *M. furfur* (1:5 *v*/*v*) were performed in specific media (i.e., Roswell Park Memorial Institute-RPMI for Candida spp. and Sabouraud Dextrose broth—SAB + 1% Tween 80 for *M. furfur*). One hundred microliters of the final dilution were transferred into a 96-well microtiter plate. Serial 1:2 dilutions of EOs ranging from 0.015 to 20 µL/mL were added to the wells of a 96-well plate (100 μL/well). The MIC end point was defined as the lowest concentration that produced a prominent decrease in turbidity (100%) relative to that of the drug-free control.

The MFC was measured by taking 100 μL of cell suspension from each well after 48 h (for *Candida* spp.) or 72 h (for *M. furfur*) of incubation at 32 °C, and then they were centrifuged, washed three times with fresh medium and vortexed for 10s. The solution was cultured on a specific medium (SDA for *Candida* spp. and SDA + 1% Tween 80 for *M. furfur*) at 32 °C for 72 h. The MFC value was defined as the MIC values of drugs at which no visible growth was detected. The MIC and MFC values of fluconazole (FLZ) were also detected as positive controls.

The negative control was yeast in broth without any antifungal. The experiment was repeated in duplicate three times on different days. Data obtained were reported as MIC ranges and MIC_90_ which indicate EO or drug concentration that inhibits the growth of 90% of the isolates. 

### 4.4. Inhibitory Effects of Cymbopogon citratus and Cymbopogon proximus EOs on Candida *spp.* and Malassezia furfur Biofilms

The biofilm reduction of *Candida* spp. and *M. furfur* by *C. citratus* and *C. proximus* EOs was evaluated according to a previously reported method [58]. *Candida* spp. and *M. furfur* biofilms were performed in microtiter plates by adding 100 µL of cell suspension (1 × 106 cells/mL) suspended in RPMI 1640 medium (*Candida* spp.) and in SAB supplemented with 1% Tween 80 (*M. furfur*) and incubated at 37 °C for 24 h for *Candida* spp. and 48 h for *M. furfur*. The wells were then washed twice with sterile phosphate buffered saline (PBS) and 100 µL of RPMI or/and SAB tween 1% containing *C. citratus* (10 and 20 µL/mL) or *C. proximus* EOs (80 and 40 µL/mL), or FLZ (16 and 8 µg/mL) were added. A medium (100 µL) without EOs was used as a negative control for biofilm growth. Microtiter plates were incubated at 37 °C for an additional 24 h for *Candida* spp. and 48 h for *M. furfur*. Then, the medium was removed, and the wells were washed twice with sterile PBS (200 mL per well). Semi-quantification of the fungal cell viability in wells of microtiter plates was calculated using a colorimetric XTT [2,3-bis (2-methoxy-4-nitro-5-sulfo-phenyl)-2H-tetrazolium-5-carboxanilide reduction assay. XTT (Sigma-Aldrich, Milan, Italy) was prepared in a saturated solution at 0.5 g/L in PBS. The solution was filter sterilized with a filter with a pore size of 0.22 mm, aliquoted and stored at −80 °C. Prior to each assay, an aliquot of stock XTT was supplemented with menadione (10 mM stock to a final concentration of 1 µM; Sigma-Aldrich, Milan, Italy). 100 µL of XTT–menadione solution was added to each pre-washed biofilm and control well. The microtiter plates were incubated in the dark at 37 °C for 3 h. Following incubation, 80 µL of the resulting-colored supernatant was transferred to a new microtiter plate and the colorimetric change from XTT reduction was read in at 490 nm using a microtiter plate reader (Benchmark Microplate Reader; Bio-Rad, Hercules, CA, USA). The results were reported as a percentage of biofilm inhibition using the following formula: % inhibition = [(control OD_490_ nm − Test OD_490_ nm)/control OD_490_ nm] × 100.

### 4.5. In Vitro Antioxidant Activity of C. citratus and C. proximus EOs

The radical scavenging activity of the EOs against 2,2-Diphenyl-1-(2,4,6-trinitrophenyl) hydrazy (DPPH) was determined as previously reported [59]. Briefly, 1 mL of EO solution in methanol (Sigma-Aldrich, Milan, Italy) ranging from 0.5 to 70 μg/mL was combined with 2 mL of methanol DPPH solution (0.1 mM). The obtained samples were mixed vigorously and kept in the dark for 60 min. The absorbance was measured at 517 nm using a double beam UV-VIS spectrophotometer (Shimadzu UV-1601, Kyoto, Japan). Methanol was used as a negative control. Ascorbic acid and Trolox were used as positive controls.

The percentage inhibition of the DPPH radical was calculated according to the following formula: % Inhibition = [(control OD − sample OD)/control OD] × 100, where A is absorbance at 517 nm. The results were expressed as of EC_50_ (μg EO /mL), which is the concentration necessary to obtain a 50% reduction of DPPH^•^ radical.

The 2,2′-Azino-Bis-3-Ethylbenzothiazoline-6-Sulfonic Acid (ABTS) radical scavenging activity of EOs was determined as previously reported [60]. ABTS^+^ was generated by the reaction of a 7 mM aqueous solution of ABTS with 2.45 mM aqueous solution of K_2_S_2_O_8_ which was conducted in the dark at room temperature for 16 h before use. The ABTS^+^ solution was diluted with ethanol to an absorbance of 0.70 (±0.02) at 734 nm. About 0.15mL of different concentrations of EOs was mixed with 2.85 mL of ethanolic solution of ABTS^+^, and the absorbance was read at 734 nm using a spectrophotometer after 15 min. Ethanol was used as a negative control. Ascorbic acid and Trolox were used as positive controls. The ABTS^+^ inhibition radical was calculated according to the following formula: % inhibition = [(control OD − sample OD)/control OD] × 100, where A is the absorbance at 734 nm. The results were expressed in terms of EC_50_ (μg EO/mL), which is the concentration necessary for 50% reduction of ABTS^+^ radical. EC_50_ was calculated from the graph plotting the percentage of radical scavenging activity (DPPH or ABTS) against EO concentration (1.5, 2.5, 5, 10, 20, 30, 50 and 70 µg/mL).

### 4.6. Statistical Analysis

The statistical analyses were performed using the Statistical Package for the Social Sciences (SPSS) for Windows program (version 13.0, SPSS Inc., San Rafael, CA, USA). One-way analysis of variance (one-way ANOVA) with post-hoc Tukey HSD (Honestly Significant Difference) was used to compare the differences among the MIC, EC_50_, biofilm optical densities of different yeast species, and the percentage of biofilm inhibition of *C. citratus* and *C. proximus* EOs. Differences were considered statistically significant when *p* < 0.05.

## 5. Conclusions

Conclusively, this study suggests that *C. citratus* and *C. proximus* EOs could be considered an excellent source of pharmacology ingredients to treat aging-associated diseases caused by free radicals for their antioxidant activities and to treat or prevent fungal infections and in particular might be considered as a drug source for preventing long treating *Malassezia* spp. skin infections in both medical and veterinary medicine. This study confirms the potential benefits of the use of natural antioxidants as antibiofilm compounds. Further investigations on the mechanism of action of antioxidant agents in treating, preventing and eradicating fungal biofilm are required.

## Figures and Tables

**Table 1:** The main constituents of *Cymbopogon citratus* EO.

Peak No.	RT	Name	Formula	Classification	MS (M/e)				Area %
					m/z	No Scans	Main Significant Fragments	Base Peak	
1	10.133	β-Myrcene	C_10_H_16_	monoterpenes	136.23	18	69, 79, 93, 121	93	5.82
2	14.046	Linalyl acetate	C_12_H_20_O_2_	monoterpenes	196.29	52	55, 69, 79, 93, 107, 121, 136, 150	93	0.58
3	16.289	trans-Verbenol	C_10_H_16_O	monoterpenoid alcohol	152.23	51	55, 67, 91, 109, 134	91	1.01
4	16.93	Isoneral	C_10_H_16_O	monoterpenes	152.23	51	55, 67, 81, 91, 109, 119, 134, 152	67, 81	1.26
5	19.082	Z-Citral B (Neral)	C_10_H_16_O	monoterpenes	152.24	45	69, 94, 134	69	37.49
6	19.568	Nerol	C_10_H_18_O	monoterpenoid alcohol	154.25	40	69, 79, 93, 121, 154	69	3.65
7	20.163	E-Citral A (Geranial)	C_10_H_16_O	monoterpenes	152.23	53	69, 84, 109, 152	69	48.2
8	23.499	Grandlure II	C_10_H_18_O	monoterpenes	154.25	75	55, 69, 79, 93, 121, 136, 154	69	1.91
9	24.993	trans-α-Bergamotene	C_15_H_24_	bicyclic sesquiterpenoids	204.35	20	55, 69, 79, 93, 107, 119, 135, 161	93	0.07
Total Identification			99.99						
Total monoterpenes			99.92						
Total sesquiterpenes			0.07						

**Table 2:** The main constituents of *Cymbopogon proximus* EO.

Peak No.	RT	Name	Formula	Classification	MS (M/e)				Area %
					m/z	No Scans	Main Significant Fragments	Base Peak	
1	10.327	α-Terpinolene	C_10_H_16_	monoterpenes	136.23	18	93, 120	93	15.7
2	11.26	cis-β-terpinyl acetate	C_12_H_20_O_2_	monoterpenes	196.28	22	68, 93	93	2.91
3	17.085	α-Terpineol	C_10_H_18_O	monoterpenoid alcohol	154.25	54	59, 93	93	1.44
4	19.414	piperitone	C_10_H_16_O	monoterpenes	152.23	52	69, 82, 109	82	66.99
5	28.249	β-Elemol	C_15_H_26_O	sesquiterpenes	222.37	52	59, 93, 161	59	5.87
6	30.818	Selinenol	C_15_H_26_O	sesquiterpenes	222.37	23	91, 133, 189	189	1.56
7	31.516	β-Eudesmol	C_15_H_26_O	sesquiterpenes	222.37	23	59, 91, 149, 204	59	2.42
8	31.636	γ-Eudesmol	C_15_H_26_O	sesquiterpenes	222.37	23	59, 91, 149, 204	59	3.11
Total Identification			100						
Total monoterpenes			87.04						
Total sesquiterpenes			12.96						

**Table 3:** Minimum inhibitory concentration (MIC) and minimum fungicidal concentration (MFC) of *Cymbopogon citratus* and *Cymbopogon proximus* EOs and fluconazole (FLZ) against *Candida* spp. and *Malassezia furfur*.

Yeast spp.	MIC Values	*C. citratus* EO		*C. proximus* EO		FLZ	
		MIC µL/mL	MFC µL/mL	MIC µL/mL	MFC µL/mL	MIC µL/mL	MFC µL/mL
*Candida tropicalis* (*n* = 7)	Range	2.5	2.5	20	<20	4	4
	MIC_90_	2.5	2.5	20	<20	4	4
*Candida catenulate* (*n* = 10)	Range	2.5–5	5	20	<20	8	8
	MIC_90_	5	5	20	<20	8	8
*Candida krusei* (*n* = 10)	Range	2.5	2.5	20	<20	>32	>32
	MIC_90_	2.5	2.5	20	<20	>32	>32
*Candida guilliermondii* (*n* = 10)	Range	2.5–5	5	20	<20	8	8
	MIC_90_	2.5	5	20	<20	8	8
*Candida albicans* (*n* = 12)	Range	2.5	2.5	20	<20	4	4
	MIC_90_	2.5	2.5	20	<20	4	4
*Malassezia furfur* (*n* = 9)	Range	1.25	2.5	2.5	2.5	>32	>32
	MIC_90_	1.25	2.5	2.5	2.5	>32	>32
*Candida parapsilosis* (*n* = 8)	Range	2.5	2.5	20	<20	4	4
	MIC_90_	2.5	2.5	20	<20	4	4
*Candida parapsilosis* ATCC 22019	Range	2.5	2.5	20	<20	4	4
	MIC_90_	2.5	2.5	20	<20	4	4
*Candida krusei* ATCC 6258	Range	2.5	2.5	20	<20	>32	>32
	MIC_90_	2.5	2.5	20	<20	>32	>32

**Table 4:** Percentage of *Candida* spp. and *Malassezia furfur* biofilm inhibition by *C. proximus* and *C. citratus* EOs at different concentrations.

	*C. tropcalis* (*n* = 7)	*C. catenulata* (*n* = 10)	*C. krusei* (*n* = 10)	*C. guillermondii* (*n* = 10)	*C. albicans* (*n* = 12)	*M. furfur* (*n* = 9)	*C. parapsilosis* (*n* = 8)	*C. krusei* ATCC 6258	*C. parapsilosis* ATCC 22019
EO *C. proximus* (80 µL/mL)	71.56 ± 22.5 ^a^	43.14 ± 17.8 ^a,d^	64.86 ± 22.7 ^a,d^	77.36 ± 7.4 ^a,d^	35.94 ± 19.0 ^a^	64.47 ± 11.9 ^a^	78.77 ± 1.9 ^a^	94.96 ± 1.9 ^a^	78.77 ± 8.8 ^a^
EO *C. proximus* (40 µL/mL)	67.82 ± 12.8 ^b,c^	33.14 ± 18.5 ^b,c^	54.73 ± 19.3 ^b,c^	83.82 ± 16.5 ^b,c^	27.65 ± 11.7 ^b^	77.36 ± 8.2 ^b^	85.44 ± 20.7 ^b^	91.32 ± 3.9 ^b^	85.44 ± 2.0 ^b^
EO *C. citratus* (20 µL/mL)	90.42 ± 3.2 ^a,c^	83.64 ± 10.6 ^a,b^	96.39 ± 2.8 ^a,b^	93.64 ± 1.5 ^a,b^	94.85 ± 3.6 ^a,b^	74.01 ± 11.5 ^c^	93.68 ± 26.6 ^c^	95.32 ± 1.4 ^c^	93.68 ± 1.4 ^c^
EO *C. citratus* (10 µL/mL)	86.99 ± 5.4 ^b^	83.86 ± 10.3 ^c,d^	95.50 ± 2.5 ^d,c^	92.73 ± 2.0 ^c,d^	93.79 ± 4.30 ^c,d^	84.21 ± 5.1 ^a^	91.32 ± 24.7 ^d^	90.34 ± 0.5 ^d^	91.32 ± 1.9 ^d^
FLZ (16 µL/mL)	45.48 ± 3.4 ^a,b^	57.22 ± 5.3 ^a,b^	27.70 ± 15.3 ^a,b^	49.16 ± 7.0 ^a,b^	52.97 ± 5.9 ^a,b^	51.99 ± 15.1 ^a,b,c^	44.42 ± 25.6 ^a,b,c,d^	19.68 ± 13.1 ^a,b,c,d^	44.42 ± 6.8 ^a,b,c,d^

Statistically significant differences were reported with the same superscript letters within the column.

**Table 5:** Yeast strains used in this study.

Yeast Species	Collection Code	Origins
*Candida tropicalis* (*n* = 7)	CD1693, CD1694, CD 1695, CD1700, CD1701, CD1702, CD1703	Lizards feces
*Candida catenulata* (*n* = 10)	CD 1777, CD1778, CD1568, CD1569, CD1575, CD1577, CD1578, CD1579, CD1580, CD1581	Lizards, Laying hens feces
*Candida krusei* (*n* = 10)	CD 1631, CD 1635, CD1638, CD 1641, CD1642, CD 1645, CD 1650, CD 1651, CD 1659, CD 1661, CD1662	Wild boars feces
*Candida guilliermondii* (*n* = 10)	CD 1606, CD 1644, CD 1653, CD 1675, CD1676, CD 1733, CD1738, CD1740, CD 1741, CD1743	Lizards and wild boars feces
*Candida albicans* (*n* = 12)	CD1601, CD1613, CD1616, CD1618, CD1620, CD1637, CD 1721, CD1729, CD1730, CD1755, CD1757, CD1760	Lizards and wild boar feces
*Malassezia furfur* (*n* = 9)	CD 1008, CD1009, CD1029, CD1030 CD1042, CD1043, CD1058, CD1490, CD1492	Human skin
*Candida parapsilosis* (*n* = 8)	CD1679, CD1681, CD1682, CD1683, CD1684, CD1691, CD1735, CD1736	Lizards and wild boar feces
*Candida krusei*	ATCC 6258	American Type Culture Collection
*Candida parapsilosis*	ATCC 22019	American Type Culture Collection

## Data Availability

Data are contained within the article.
